# Impact of twin boundaries on bulk elastic constants: Density-functional theory data for Young׳s modulus of Ag

**DOI:** 10.1016/j.dib.2015.03.005

**Published:** 2015-03-28

**Authors:** Tobias Klöffel, Erik Bitzek, Bernd Meyer

**Affiliations:** a Interdisciplinary Center for Molecular Materials (ICMM) and Computer-Chemistry-Center (CCC), Friedrich-Alexander-Universität Erlangen-Nürnberg (FAU), Nägelsbachstraße 25, 91052 Erlangen, Germany; b Materials Science & Engineering, Institute I, Friedrich-Alexander-Universität Erlangen-Nürnberg (FAU), Martensstraße 5, 91058 Erlangen, Germany

**Keywords:** Elastic constants, Young׳s modulus, Twin boundaries, Density-functional theory

## Abstract

Experimental and theoretical studies on nanowires have reported a size-dependence of the Young׳s modulus in the axial direction, which has been attributed to the increasing influence of surface stresses with decreasing wire diameter. Internal interfaces and their associated interface stresses could lead to similar changes in the elastic properties. In Kobler et al. [1], however, we reported results from atomistic calculations which showed for Ag that twin boundaries have a negligible effect on the Young׳s modulus.

Here, we present data of density-functional theory calculations of elastic constants and Young׳s modulus for defect-free bulk Ag as well as for bulk Ag containing dense arrays of twin boundaries. It is shown that rigorous convergence tests are required in order to be able to deduce changes in the elastic properties due to bulk defects in a reliable way.

**Specification table**

**Table:** 

Subject area	Materials Science
More specific	Elastic properties of defect-free
subject area	crystals and with twin boundaries
Type of data	Tables and graphs
How data was	Density functional theory calculations
acquired	using the periodic plane-wave code PWscf
Data format	Analyzed
Experimental factors	Not applicable
Experimental	Not applicable
features	
Data source location	Erlangen, Germany
Data accessibility	Data are available with this paper

**Value of the data**

• Twin boundaries in bulk Ag have a negligible effect on the Young׳s modulus.
• The importance of rigorous convergence tests in density-functional theory calculations of elastic properties is shown.
• The density of the *k*-point mesh and the width *σ* of the broadening scheme, which is used to determine the occupation numbers, are the most crucial parameters.

## 1 Computational methods

Density-functional theory (DFT) calculations were carried out with the plane-wave code *PWscf* of the Quantum Espresso software package [2], using the Perdew–Burke–Ernzerhof PBE exchange-correlation functional [3], Vanderbilt ultrasoft pseudopotentials [4] and a plane wave kinetic energy cutoff of 30 Ry. *k*-point meshes for Brillouin zone integrations were generated by the Monkhorst–Pack scheme [5], and the fractional occupation numbers of the electronic states were determined by a Gaussian broadening [6]. Atomic positions and lattice parameters were relaxed by minimizing the atomic forces and the stress tensor, with a convergence threshold for the largest residual force and stress component of 5 meV/Å and 0.1 kbar, respectively.

Bulk second order elastic constants (SOEC) were calculated by finite deformations of the conventional face-centered cubic (fcc) unit cell with 4 atoms, Cartesian basis vectors and lattice constant *a*. The lattice is distorted by applying a small strain $\underset{̲}{\varepsilon }$, which transforms the basis vectors $a_{1}$, $a_{2}$, $a_{3}$ to the new vectors

(1) $$({\overset{˜}{a}}_{1},{\overset{˜}{a}}_{2},{\overset{˜}{a}}_{3})=(\underset{̲}{I}+\underset{̲}{\varepsilon })(a_{1},a_{2},a_{3}),$$

where $\underset{̲}{I}$ is the 3×3 identity matrix. The three independent elastic constants of the cubic lattice *C*_11_, *C*_12_ and *C*_44_ were determined by using the deformation strains proposed by Mehl et al. [7]:

(2) $$\underset{̲}{\varepsilon }=(\begin{array}{ccc} x & 0 & 0\\ 0 & x & 0\\ 0 & 0 & x \end{array}),\;\underset{̲}{\varepsilon }=(\begin{array}{ccc} x & 0 & 0\\ 0 & -x & 0\\ 0 & 0 & \frac{x^{2}}{1-x^{2}} \end{array}),\;\underset{̲}{\varepsilon }=(\begin{array}{ccc} 0 & \frac{x}{2} & 0\\ \frac{x}{2} & 0 & 0\\ 0 & 0 & \frac{x^{2}}{4-x^{2}} \end{array}).$$

The first deformation is a homogeneous volume change, which changes the total energy of the unit cell by

(3) $$\Delta E(x)=\frac{1}{2}V_{0}{\mathrm{Bx}}^{2}+O[x^{3}].$$

*V*_0_ is the equilibrium volume of the unit cell and *B* is the bulk modulus. For a cubic lattice *B* is related to the elastic constants by

(4) $$B=\frac{1}{3}(C_{11}+2C_{12}).$$

The second deformation is a volume conserving orthorhombic strain with energy change

(5) $$\Delta E(x)=V_{0}(C_{11}-C_{12})x^{2}+O[x^{4}],$$

and the third deformation is a volume conserving monoclinic shear with energy change

(6) $$\Delta E(x)=\frac{1}{2}V_{0}C_{44}x^{2}+O[x^{4}].$$

From the bulk modulus *B* and the difference $\Delta C=C_{11}-C_{12}$ the two elastic constants *C*_11_ and *C*_12_ are given by

(7) $$C_{11}=B+\frac{2}{3}\Delta C\;\mathrm{and}\;C_{12}=B-\frac{1}{3}\Delta C.$$

For all three deformations a series of total energy calculations was performed for a small set of finite strain values *x*. The energy values as function of *x* were fitted to a polynomial and the elastic constants were determined from the second derivative of the polynomial at the minimum. Specifically, the bulk modulus *B* was calculated by varying the lattice constant *a* between 7.64 Bohr and 8.04 Bohr in steps of 0.04 Bohr, which corresponds to strain values between −2.4% and +2.7%. A polynomial of degree 4 was fitted to the 11 data points. The minimum of the curve gives the equilibrium lattice constant, which was used as starting point for the calculation of $C_{11}-C_{12}$ and *C*_44_. The deformations proposed by Mehl have the advantage that the energy is an even function of the strain *x*. Thus, only positive values for *x* have to be considered. For the calculation of $C_{11}-C_{12}$ the strain parameter *x* was changed between 0 and +0.084 in steps of +0.012 (8 values) and for the calculation of *C*_44_ we used 7 values of *x* between 0 and +0.12 in steps of +0.02. An even polynomial of degree 6 was fitted to the calculated total energy values.

The Young׳s modulus $E_{\left[\mathrm{hkl}\right]}$ was calculated by a similar quastistatic approach as the SOEC. The unit cell is chosen in such a way that one axis is parallel to the direction [hkl] of the applied strain and the two other axes are perpendicular to the first one. Then a set of finite tensile and compressive strains *x* is applied and for each strain *x* the cell vectors perpendicular to the strain direction and the atomic positions are relaxed (Poisson contraction). From the energy change

(8) $$\Delta E(x)=\frac{1}{2}V_{0}E_{\left[\mathrm{hkl}\right]}x^{2}+O[x^{3}]$$

The Young׳s modulus $E_{\left[\mathrm{hkl}\right]}$ is calculated by taking the second derivative of a polynomial fitted to the energy versus strain values.

For the calculation of the bulk value of the Young׳s modulus $E_{\left[110\right]}$ the simplest choice is a 2-atom tetragonal unit cell with cell vectors along [$1\overset{¯}{1}0$], [110], and [001]:

(9) $$a_{1}=\frac{a}{\sqrt{2}}(\begin{array}{c} 1\\ 0\\ 0 \end{array}),\;a_{2}=\frac{a}{\sqrt{2}}(\begin{array}{c} 0\\ 1\\ 0 \end{array}),\;a_{3}=a(\begin{array}{c} 0\\ 0\\ 1 \end{array}).$$

This cell, however, does not allow to introduce twin boundaries. Therefore, the calculation of $E_{\left[110\right]}$ was repeated for a second, 6-atom orthorhombic unit cell with cell vectors along [$10\overset{¯}{1}$], [$1\overset{¯}{2}1$] and [111]:

(10) $$a_{1}=\frac{a}{\sqrt{2}}(\begin{array}{c} 1\\ 0\\ 0 \end{array}),\;a_{2}=\sqrt{\frac{3}{2}}a(\begin{array}{c} 0\\ 1\\ 0 \end{array}),\;a_{3}=\sqrt{3}a(\begin{array}{c} 0\\ 0\\ 1 \end{array}).$$

The cell contains three atomic layers with ABC stacking along [111], with 2 atoms per plane in the unit cell. This cell can also be used for calculating Young׳s modulus $E_{\left[112\right]}$. In a cubic crystal, however, $E_{\left[110\right]}$ and $E_{\left[112\right]}$ are equal due to symmetry. It is important to note that for the orthorhombic unit cell the Poisson contraction gives rise to a tilt of the basis vectors $a_{2}$ and $a_{3}$, if a strain is applied in the direction of $a_{1}$. Thus, the angle between $a_{2}$ and $a_{3}$ has to be relaxed in order to get the correct value for the Young׳s modulus $E_{\left[110\right]}$.

Unit cells for the bulk crystal with twin boundaries are derived from the orthorhombic cell, but with a different number of atomic planes in the [111] direction ($a_{3}$ axis). Periodic boundary conditions require that always 2 twin boundaries are included in one unit cell. We used cells with 6, 8 and 10 atomic layers (thus containing 12, 16 and 20 atoms) with stacking sequences of ABC BAC, ABCA CBAC and ABCAB ACBAC, respectively, in which the twin boundaries are separated by $3a/\sqrt{3}$, $4a/\sqrt{3}$, $5a/\sqrt{3}=7.18\;$Å, 9.57 Å, 11.96 Å.

In all cases, the Young׳s modulus $E_{\left[110\right]}$ was calculated by changing the length of $a_{1}$ between −0.024 and +0.030 in steps of 0.06 in units of $a/\sqrt{2}$. This corresponds to applied strains between −3.4% and +4.2%. A polynomial of degree 6 was fitted to the 10 total energy values.

## 2 Results of the DFT benchmark calculations

Since elastic constants are second derivatives of the total energy, DFT calculations have to be very well converged in order to be able to extract elastic constants in a reliable way (within the limits of the accuracy of the chosen functional). First, we thoroughly tested the influence of the plane wave basis set and density cut-off energies. For our choice of these cut-off energies (30 Ry and 120 Ry, respectively), the elastic constants are well converged within ±0.1 GPa. Much more crucial is the convergence with respect to *k*-point density and Gaussian smearing parameter *σ*. An additional problem arises from the fact that different unit cells have to be used for the calculation of the bulk value of the Young׳s modulus and of structures containing twin boundaries, since *k*-point meshes will not be equivalent. Therefore, to be able to identify changes in elastic properties due to twin boundaries, absolute values of elastic constants have to be converged as good as possible. To have an estimate on how well our calculations are converged and what accuracy for changes in the elastic constants can be expected, we applied the following 3 step strategy:

(1) First, the bulk second-order elastic constants (SOEC) are calculated and we examine their convergence with respect to the *k*-point mesh and the Gaussian smearing parameter *σ*.
(2) Then we perform direct calculations of the bulk Young׳s modulus (using both, the tetragonal and orthorhombic unit cell) by quasistatic tensile tests. The results of both calculations are compared to the analytical value of the Young׳s modulus as given by the SOFCs. In cubic crystals, the Young׳s moduli $E_{\left[110\right]}$ and $E_{\left[112\right]}$ are identical and are related to the SOEC by
  (11) $$E_{\left[110\right]}=E_{\left[112\right]}=4{\left[\frac{2C_{11}}{\left(C_{11}-C_{12})(C_{11}+2C_{12}\right)}+\frac{1}{C_{44}}\right]}^{-1}.$$
  The variation between the three approaches indicates how well our calculations are converged. The deviation will be used as an estimate for the error margin that we have to take into account for our results of the Young׳s modulus.
(3) Finally, twin boundaries are introduced in the orthorhombic unit cell and quasistatic calculations of the Young׳s modulus are performed. The results are compared to the bulk values obtained in step (2).

**Fig. 1:**
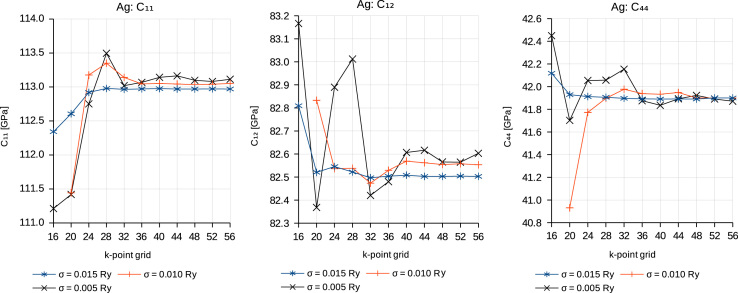
Convergence of the Ag bulk elastic constants with respect to the *k*-point mesh and the Gaussian smearing parameter *σ*. Calculations were performed with the conventional 4-atom cubic unit cell.

The results for step (1) are shown in Fig. 1 and Table 1. *k*-point grids were always of the Monkhorst–Pack type with divisions of (*n*, *n*, *n*) of the three basis vectors of the reciprocal lattice. Fig. 1 shows the typical behavior for the convergence of properties with *k*-point density *n*: the larger the Gaussian smearing parameter *σ*, the faster the *k*-point convergence, but the *k*-point-converged result depends on the value of *σ*. This is most obvious for the elastic constant *C*_12_. However, for our choices of *σ* of 0.005, 0.010 and 0.015 Ry the deviation from the *σ*=0 limit is less than 0.1 GPa for all elastic constants. Table 1 summarizes the results of the elastic constants for the different smearing values *σ* together with the *k*-point density, which is required for obtaining convergence within an accuracy of ±0.1 GPa. Obviously, for elastic properties a much denser *k*-point mesh than for the calculation of energy differences (for example, surface and interface energies) are required.

In Table 1 also the experimental values for the SOEC at 0 K and 300 K are given. The DFT calculations underestimate the elastic constant by up to 20%. This is typical for the PBE functional and other functionals based on the generalized-gradient approximation (GGA). In the local density approximation (LDA), on the other hand, elastic constants are overestimated by up to 20% [11]. However, this uncertainty of DFT calculations (the dependence of the results for SOEC on the choice of functional) is not crucial for our aim: we are not interested in the *absolute* values of elastic properties, but only in the *change* of the Young׳s modulus after the introduction of twin boundaries.

The results of the direct, quasistatic calculations of the bulk value of the Young׳s modulus $E_{\left[110\right]}$ in step (2) are summarized in Table 2. *k*-points for the tetragonal and orthorhombic unit cells were also chosen according to the Monkhorst–Pack scheme. In Table 2 the number of divisions *n*, however, refers to an approximately equivalent (*n*, *n*, *n*) *k*-point mesh for the conventional 4-atom cubic unit cell with lattice constant *a*. The lattice vectors of the tetragonal and orthorhombic unit cells have lengths of $a/\sqrt{2}$, $\sqrt{(3/2})\;a$ and $\sqrt{3}\;a$. Thus, in the actual calculation divisions of $\sqrt{2}n$, $\sqrt{(2/3})n$ and $n/\sqrt{3}$ of the corresponding reciprocal basis vector, rounded to the next larger integer number, were used.

For a Gaussian smearing of *σ*=0.015 Ry the SOEC were well converged for a *k*-point density of 28 and 24 (see Fig. 1). For this *σ*-value and the *k*-point mesh with *n*=28, however, the Young׳s modulus $E_{\left[110\right]}$ calculated by quasistatic tensile test still deviates by 1.8 GPa from the analytic value calculated from the SOEC (see Table 2). For a better agreement between direct calculation and analytic result from SOEC for $E_{\left[110\right]}$, *σ* has to be lowered to 0.010 Ry and the *k*-point density has to be increased to *n*=32. With the settings of *σ*=0.015 Ry and the *k*-point mesh of *n*=28 the error margin of the DFT calculations is more than 1 GPa, for *σ*=0.010 Ry and the *k*-point mesh with *n*=32 the numerical uncertainty is reduced to about 0.3 GPa.

In Table 3 the calculated Young׳s moduli for the orthorhombic unit cells with twin boundaries are compared to their corresponding values for the defect-free bulk. For the calculations with *σ*=0.015 Ry and the *k*-point mesh with *n*=28 the results for $E_{\left[110\right]}$ for cells with and without twin boundaries are within our estimated error margin. For these settings the unit cells with twin boundaries were also strained in [112]-direction, which is the actual orientation of the nanowires in the experiments of Ref. [1], but no significant difference between $E_{\left[110\right]}$ and $E_{\left[112\right]}$ is observed.

For the more accurate settings with *σ*=0.010 Ry and a *k*-point mesh with *n*=32, the Young׳s modulus $E_{\left[110\right]}$ is systematically smaller for cells with twin boundaries than the bulk value, and $E_{\left[110\right]}$ decreases systematically with increasing twin boundary density. However, even for the highest density of twin boundaries, in which twin boundaries are separated by only 3 atomic planes, the change in $E_{\left[110\right]}$ is less than 2% compared to the bulk. This can be taken as an upper limit for the modification of Young׳s modulus due to twin boundaries in Ag.

## Figures and Tables

**Table 1:** *k*-point density required for a given Gaussian smearing parameter *σ* for obtaining converged second order elastic constants within 0.1 GPa for bulk Ag.

*σ* (Ry)	*k*-points	*a* (Bohr)	*C*_11_ (GPa)	*C*_12_ (GPa)	*C*_44_ (GPa)
0.015	28	7.8290	113.0	82.5	41.9
0.010	32	7.8287	113.1	82.5	42.0
0.005	40	7.8287	113.1	82.6	41.9

Exp [8,9], 300 K		7.7183	124.0	94.0	46.5
Exp [8,10], 0 K		7.6902	131.5	97.3	51.1

**Table 2:** Results for the direct quasistatic calculation of the Young׳s modulus $E_{\left[110\right]}$ for bulk Ag using either the tetragonal or orthorhombic unit cell. Values are compared to the analytic result from the SOEC. Differences are more than 1 GPa (*σ*=0.015 Ry, *k*-point mesh with *n*=28) and about 0.3 GPa (*σ*=0.010 Ry, *k*-point mesh with *n*=32). This represents a good estimate for the numerical accuracy of the calculated Young׳s modulus.

Unit cell	*k*-points	*σ* (Ry)	$E_{\left[110\right]}$ (GPa) quasistatic	$E_{\left[110\right]}$ (GPa) from SOEC
Tetragonal	24	0.015	81.7	79.0
Tetragonal	28	0.015	81.0	79.2
Tetragonal	32	0.010	79.7	79.4

Orthorhombic	24	0.015	81.6	79.0
Orthorhombic	32	0.010	79.2	79.4

**Table 3:** Results for the direct quasistatic calculation of the Young׳s moduli $E_{\left[110\right]}$ and $E_{\left[112\right]}$ for the orthorhombic unit cell with two twin boundaries.

Layers	*k*-points	*σ* (Ry)	Twin: $E_{\left[110\right]}$ (GPa)	Twin: $E_{\left[112\right]}$ (GPa)	Bulk: quasistatic $E_{\left[110\right]}$ (GPa)	Bulk: from SOEC $E_{\left[110\right]}$ (GPa)
6	28	0.015	80.5	79.7	81.0	79.2
8	28	0.015	80.6	79.9	81.0	79.2
10	28	0.015	80.3	79.8	81.0	79.2

6	32	0.010	77.9	–	79.2	79.4
8	32	0.010	78.2	–	79.2	79.4
10	32	0.010	78.3	–	79.2	79.4
